# Alpha-1 Antitrypsin Deficiency PI*Z and PI*S Gene Frequency Distribution Using on Maps of the World by an Inverse Distance Weighting (IDW) Multivariate Interpolation Method

**DOI:** 10.5812/hepatmon.7434

**Published:** 2012-10-21

**Authors:** Ignacio Blanco, Frederick J. de Serres, Victoriano Cárcaba, Beatríz Lara, Enrique Fernández-Bustillo

**Affiliations:** 1 The Principality of Asturias Biomedical Research Office (OIB-FICYT), Oviedo, Spain; 2 Alpha1-Antitrypsin Deficiency Spanish Registry, Barcelona, Spain; 3 National Institute of Environmental Health Sciences, Research Triangle Park, NC, USA; 4 Department of Internal Medicine, ‘Valle del Nalón Hospital, Principado de Asturias, Spain; 5 Hospital Universitario Arnau de Vilanova, Avda, Institut de Recerca Biomédica de Lleida (IRB), CIBERES Instituto Salud Carlos III, Pneumology Service, Institut de Recerca Hospital Universitari Vall d’Hebron, Barcelona, Spain; 6 Biostatistics Unit, Central University Hospital of Asturias, Principado de Asturias, Spain

**Keywords:** Alpha 1-Antitrypsin, Alpha 1-Antitrypsin Deficiency, Molecular Epidemiology

## Abstract

**1.Background:**

Currently, there is a remarkable lack of genetic epidemiological studies on alpha 1-antitrypsin (AAT) deficiency in about half of the 193 countries of the World. This fact impedes the establishment of a true prevalence pattern of this deleterious hereditary disorder in extensive regions of human population.

**2.Objectives:**

The aim of the present study was to generate detailed maps of the frequency distribution of the two most frequent AAT deficiency alleles (i.e., PI*S and PI*Z) in all areas of the World.

**3.Materials and Methods:**

Available data provided by epidemiological studies performed in 94 of 193 countries worldwide was used to develop detailed maps of these two alleles, We employed an informatics mathematical approach, namely: the ArcMap [a component of ESRI’s ArcGIS Geographical Information System (GIS), for Microsoft Windows], based on the inverse distance weighting (IDW) multivariate interpolation method, which creates new numerical points from known data, using a simple logarithm based in the distance existing between them

**4. Results:**

In this method, PI*S and PI*Z frequencies were represented by colored scales, where qualitative colors were converted into quantitative data, providing information on their distribution in all parts of the world. This approach not only confirmed our previous data, but also provided digital images of the remaining regions of all continents.

**5.Conclusions:**

By using this approach, striking differences were found among regions, and unsuspected significant values of the PI*S and PI*Z alleles frequencies were obtained for several geographic regions where have not been studied yet. In fact, some of these regions might be considered as priority targets for further screening studies on AAT deficiency, in order to identify, and properly manage, individuals at risk for the diverse adverse health effects associated with AAT deficiency.

## 1. Background

Alpha1-antitrypsin (AAT) deficiency is a recessive hereditary metabolic disorder which results in the synthesis and secretion of defective AAT. Up to now, about 100 genetic variants of AAT have been recognized. Protease inhibitor (PI) M (medium mobility) is the normal allele, while the two most frequent deficient alleles are PI*S and PI*Z. The PI*ZZ genotype results in very low AAT serum concentrations (10–15%), and PI*SZ and PI*MZ phenotypes result in low to intermediate serum AAT concentrations from 35% to 70%. Other 30 variants affect the amount or the function of the AAT molecule, but clinically, most (96%) AAT deficiency-related patients are linked with the PI*Zz type, and the remaining 4% to PI*SZ, null and about other 30 rare or null phenotypes (1, 2). Individuals who are homozygous for the Z allele undergo significant intracellular polymerization of their AAT, showing a profound suppression of their circulating plasma AAT levels. The retained AAT polymers in the endoplasmic reticulum of hepatocytes can cause liver damage, whilst the lack of circulating protein may promote development of COPD (3). The presentation of patients with severe AAT deficiency differs from chronic liver disease, fulminant hepatic failure or adult emphysema (1-4). In infancy, the typical presentation is a neonatal cholestasis. Population-based studies indicate that 80% of these infants are healthy and free of chronic disease by the age of 18 years, and that the overall risk of life-threatening liver disease in childhood may be as low as 3%, but that the risk of varying degrees of liver dysfunction in children may range from 15% to 60%. Liver disease in adults may present as chronic hepatitis, and the risk of clinically significant disease may increase with advancing age (cirrhosis may be present in 30% to 40% of elderly adults, as shown by autopsy studies). There is also an increased risk of hepatocellular carcinoma of unknown magnitude in PI*ZZ adults. Other uncommon AAT deficiency-related diseases are fibromyalgia, systemic vasculitis, relapsing panniculitis and bronchial asthma (5-8). In previous epidemiological studies, data on genetic AAT deficiency from 94 of the 193 countries worldwide were published in the peer-reviewed medical literature (9-16), most of these belong to developed countries from Europe, North America, Australia and New Zealand. However, there is an important lack of AAT deficiency epidemiological data from many other regions of the World, rises the erroneous concept that this genetic disorder is either very rare or does not exist in these unexplored geographic regions (17). Thus, to assess the AAT deficiency allelic distribution in these non-studied regions of the World, we used a useful method usually employed in other disciplines of the Science (such as Geography, Meteorology, etc.), known as inverse distance weighting (IDW) multivariate interpolation (18, 19).

## 2. Objectives

From the available data on PI*S and PI*Z AAT deficiency gene frequencies, colored maps introducing new data on PI*S and PI*Z frequencies were obtained in each region and country of the World.

## 3. Materials and Methods

### 3.1. Source of Genetic Epidemiological Studies for PI*S and PI*Z Frequencies

The articles used in the present study were obtained through a variety of sources which have been discussed in earlier publications (9, 11-16). The complete database comprises a total of 514 cohorts, containing 199,449 subjects from 94 countries from Europe, America, Asia, Australia and New Zealand. These 94 countries are only those from a possible 193 countries worldwide (https://www.cia.gov/cia/publications/factbook/index.html) where there are genetic epidemiological studies available on AAT deficiency in the peer-reviewed medical literature. 

### 3.2. Inverse Distance Weighting (IDW) Multivariate Interpolation Method

In general, interpolation is a method of constructing new data points within the range of a set of known data points obtained by sampling or experimentation. Inverse distance weighting (IDW) is a process of assigning values to unknown points by using values from scattered set of known points. In a digital image (or bitmap) each pixel has a value to construct an image (18, 19). According to how far each grid point is away from the original center of each pixel, the new sample point is given a color value. The mathematics underlying bitmap is multivariate interpolation in two spatial variables, and is performed separately for each color channel. To elaborate colored geographical maps, The ArcMap “Geostatistical Analyst”[a component of ESRI’s ArcGIS Geographical Information System (GIS)], developed as client software specifically for the Microsoft Windows environment, was employed to enable more intuitive processing and presentation of ArcGIS data. The “Geostatistical wizard” option of the ArcMap menu automatically performed a “nearest point method” local interpolation IDW, where a sample point takes the four closest pixel centers and linearly interpolates their color values according to their distances from the sample point. To show the results of the interpolation graphically, the “Contours” (isolines) from the “Shape type” options was selected. To express the range of values in the World maps, a progressive 15 color scale, with blue tones representing the lowest values and red tones the highest ones, was used. The large numbers of data existing from Europe allowed to obtain a more detailed map of this continent using a 20 blue to red color grading scale.

## 4. Results

**Figure 1:**
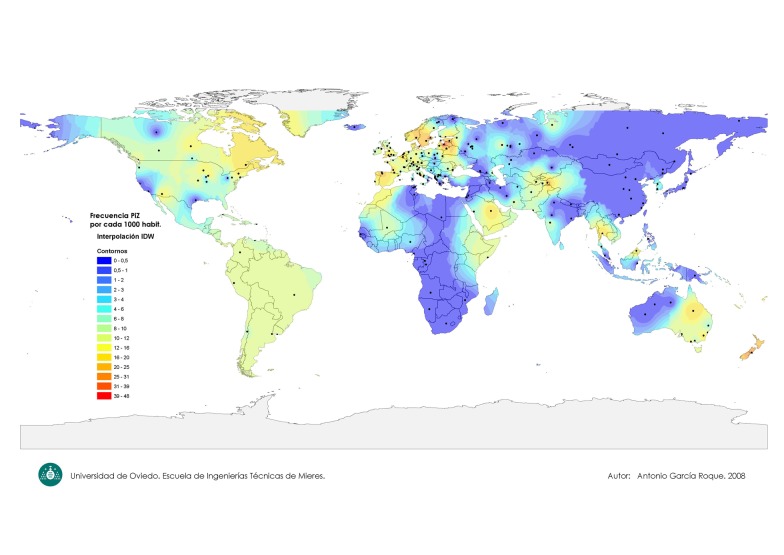
World Map of the PI*Z Gene Frequency Distribution

**Figure 2:**
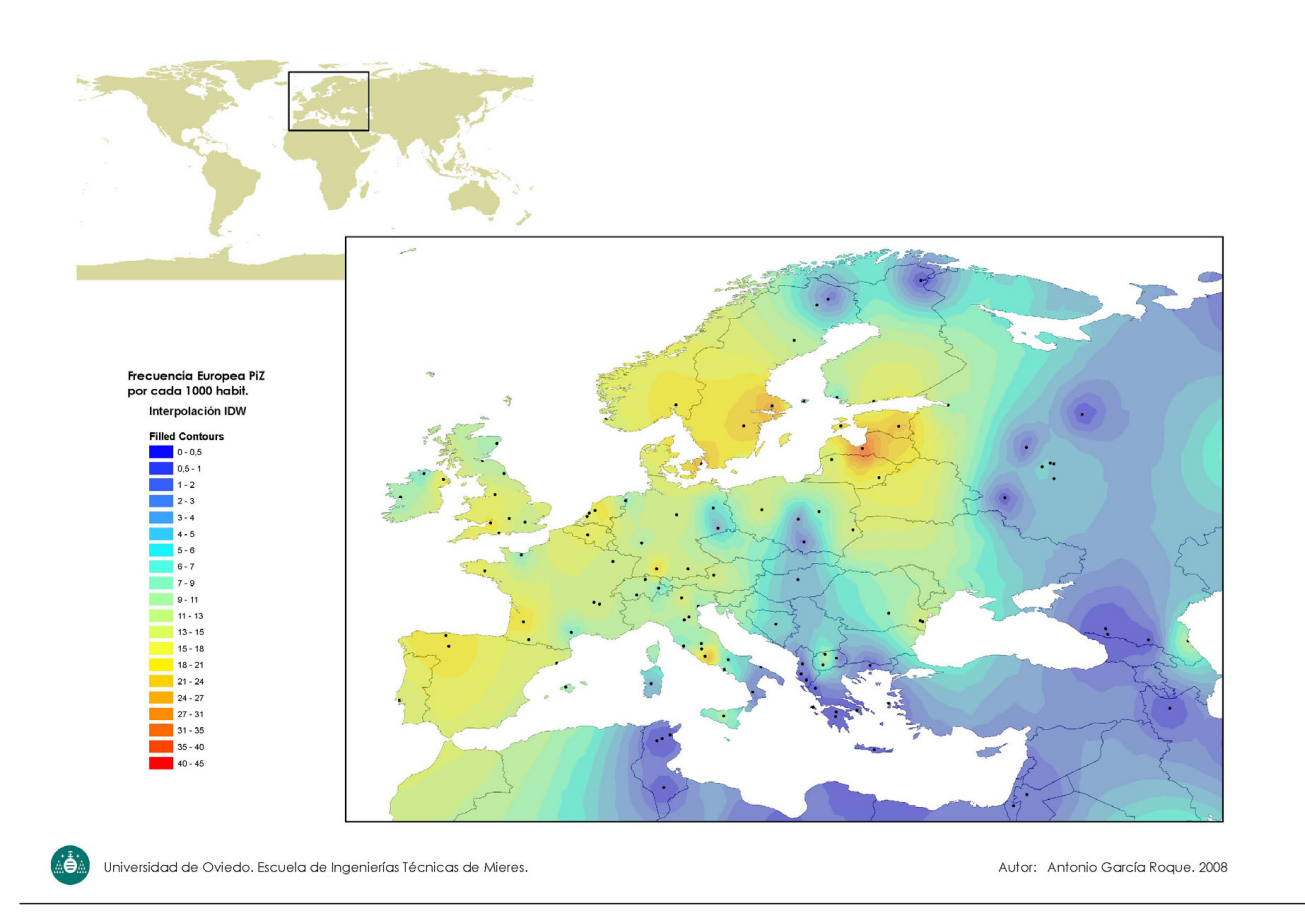
Europe Map of the PI*Z Gene Frequency Distribution

**Figure 3:**
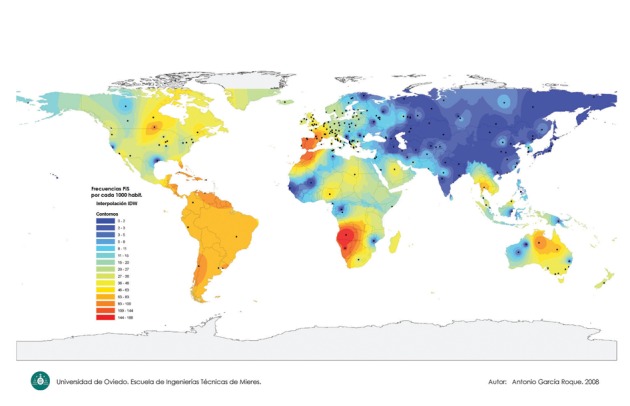
World Map of the PI*S Gene Frequency Distribution

Figure 1 shows a worldwide map indicating the PI*Z gene frequency distribution. Figure 2 focuses on the PI*Z prevalence distribution in Europe. Figure 3 shows the PI*S frequency distribution worldwide.

### 4.1. Worldwide Map of PI*Z Gene Frequency Distribution

In Figure 1, black spots indicate the places where the epidemiological studies were conducted, and where the numerical data were obtained. A colored scale with shades of red and orange tones representing maximal values (21-40 x 1000), shades of yellow and green tones representing the intermediate ones (8-20 x 1000), and shades of blue tones representing the minimal values (0-8 per 1000) has been shown. The greatest number of black spots is located in Europe (Figure 2). In contrast, spots are scattered and scanty in most of the remaining parts of the World. In general, maximal PI*Z frequencies are in European coastal regions near the Atlantic Ocean and its seas, and they gradually decrease to the west of this continent. Specifically, maximal PI*Z frequencies (20-40 per 1000) are found in the southern regions of the Scandinavian Peninsula, Denmark and Baltic Republics. Lower but still significantly high values of around 20 x 1000 are found in the Low Countries (i.e., Belgium, the Netherlands, Luxemburg, and parts of northern France and western Germany). Similar values have been found in the west coast of France, southern England, Ireland, southern regions of Great Britain, and northwestern regions of the Iberian Peninsula. Isolated points of high Z frequencies are also found in both the Trentino-Alto Adige/Südtirol and Lazio regions of Italy, and in the southern Germany states of Bavaria and Baden-Württemberg. In general, Z prevalence steadily decreases from the west to the east of the Continent, showing moderate values in some Central and Western Europe areas of Germany, Poland, Byelorussia, west of Ukraine, Macedonia, and the Black Sea coasts of Rumania and Bulgaria. PI*Z frequencies are low or very low in European Russia, Balkan Peninsula, Georgia, Armenia, Turkey, and in remote areas of the northern Europe, practically disappearing in Lapland, and the southwestern regions of the Middle East. The PI*Z allele is practically absent in Northern and Eastern Asia. However, in several regions of the Middle-East (i.e., Saudi Arabia), Southern Asia (i.e., Iran, Pakistan, Afghanistan, Tajikistan, and the coastal enclaves on the Arabian Sea coast, Daman and Diu, in Northwest India), Southeastern Asia (Thailand) and Maritime Southeast Asia (Malaysia) moderate values of the PI*Z frequency were found. Some regions of Eastern Africa (such as: Ethiopia, Somalia and Kenya) and Western Africa (i.e., Morocco, Mauritania and Mali) also showed moderate frequencies of PI*Z allele. In the remaining regions of the African Continent the PI*Z frequency is very low or absent. Moderate and high frequencies of PI*Z were found in New Zealand, and in the Australian states of Queensland, New South Wales, Victoria, South Australia and Tasmania. In the Americas, high and moderate PI*Z frequencies were found in the southwestern coast of Greenland, in several regions of Canada (i.e., British Columbia, Quebec, Ontario, New Brunswick, Prince Edward Island, Nova Scotia, Newfoundland and Labrador). In North America, high PI*Z frequencies were shown in those States located around the Great Lakes area, and also in Arizona. Moderate or low frequencies were found in the remaining zones of the U.S., and in Mexico. Moderate or low frequencies of 8-10 × 1000 were found in Central America, Caribbean Islands, and in most regions of South America. 

### 4.2. Distribution of PI*S Gene Frequencies Worldwide

In Figure 3 again the black spots indicate the sources of the data. A colored scale, with shades of red and orange tones representing maximal values (63-188 × 1000), shades of yellow and green tones the intermediate ones (20-62 × 1000), and shades of blue tones the minimal values (0-20 per 1000), has been shown. Once more, the greatest number of studies was performed in Europe, while scanty and scattered data in many other remaining geographical areas was observed. Maximal PI*S frequencies were found both in Africa and Europe. Very high PI*S frequencies were found in the Iberian Peninsula, and south-western France in accordance with previous reports. Values slowly decrease to the north and the east of the European continent. The PI*S allele is practically absent in Asia, except in some isolated areas of Saudi Arabia, Thailand and Malaysia. Surprisingly, the IDW map also showed unsuspected very high frequencies in extensive regions of Africa, with the highest PI*S frequencies worldwide found in the coastal regions of Middle and Southern Africa, corresponding to the political states of Angola, Namibia, Gabon, Congo, Botswana, and South Africa. Very high frequencies were also found in the northwestern regions of Morocco, Western Sahara and Mauritania. High PI*S frequencies were also found in other several countries of Western, Northern, Middle and Eastern Africa regions, such as: Nigeria, Chad, Sudan, Libya, Uganda and Madagascar. In the Americas, high values of PI*S were found in the Ontario province of Canada, the Florida Peninsula in the United States, several areas of Central America, the Caribbean islands, and the South American countries of Venezuela, Chile and Uruguay. In Greenland, the remaining regions of Canada, U.S.A, Mexico, and South-America, intermediate values of PI*S frequency were found. Finally, moderate values of PI*S were found in New Zealand, and high or moderate values in the Australia regions of Queensland, New South Wales, Victoria, South Australia and in Tasmania.

## 5. Discussion

AAT deficiency is an underdiagnosed disorder, partly because it is thought to be a rare condition which practically only affects Caucasians (Whites) of Northern European heritage (17). Unfortunately, at present the only data exists, has resulted from genetic epidemiological studies on AAT deficiency in 94 of the 193 countries worldwide. However, it is suspected that such genetic epidemiological studies have only “scratched the surface” due to the lack of extensive genetic epidemiological studies in countries as well as remaining areas of the World. For a first time, the informatics mathematical application ArcMap/ IDW interpolation method has provided series of colored maps, where qualitative colors were converted into quantitative data for the two more frequent AAT deficiency alleles (namely, PI*S and PI*Z) in all parts of the World. IDW interpolation consists of creating new points of values from known data, using a simple logarithm based in the distance existing between them, and currently it is a useful technique in Cartography, Topography, Meteorology, and some others. It is usually used to estimate human population distributions, environment pollution, trends and ranges of temperatures, precipitations, snowfalls, fogs, atmospheric pressures, wind velocity, frequency of days with rainfalls, relative humidity, hours of sunshine, days with frost, and some other factors. The results of the present approach not only confirmed previously reported data in countries where epidemiological data on AAT deficiency where obtained by studies performed “in situ” with real subjects, but also expanded the existing information to non-studied zones from these same countries. Likewise, the IDW interpolation method provided an estimation of the gene prevalence of the PI*S and PI*Z alleles in extensive zones of the World where epidemiological studies have not been conducted so far. Interestingly, this approach revealed the existence of intermediate and high values of both deficient alleles in some of these countries where real genetic epidemiological data is lacking. In the IDW interpolation method, the weight (value) of the points is only assigned according to the distances between them, and gives a bigger weight to the values nearer to the true point than to the more distant ones. Therefore, since in our study both the data numbers and the distances between points were much more abundant and compact in Europe, the application of a local interpolation has provided more precise maps for this continent than for the remaining ones. In contrast, in many other geographical areas outside Europe, the number of the data points obtained by direct measurement was evidently lower and with longer distances among many of them. Thus, this fact could consequently decrease the prognostic reliability of our results. Nevertheless, in spite of these possible biasing facts, and taking into account all the aforementioned limitations, the present analysis has provided guiding estimation of the PI*S and PI*Z frequency values, and has clearly demonstrated that these two major AAT deficient alleles are in widespread distribution worldwide, and that there are substantial populations at risk for adverse health effects of AAT deficiency-related in many countries of the World. These new data also indicates that AAT deficiency may constitute one of the most frequent human genetic diseases, and in most countries, individuals with phenotypes that may put them at risk for various environmental exposures have not been identified. Identification of these individuals with AAT deficiency seems to be critical for their management, education and treatment. In summary, the IDW interpolation has demonstrated to be a useful method for obtaining some information about AAT deficiency gene distribution and possible numbers of deficiency subjects in regions lacking real data on this subject. Evidently, this information might be improved by means of further epidemiological studies in countries lacking studies on AAT deficiency. Moreover, IDW interpolation could also provide valuable information on other different epidemiological aspects and disciplines of the medicine. In our study, its application specifically showed that AAT deficiency is widespread throughout the world, and that it is clear that it is not just a disease of Caucasians from Northern Europe, while is prevalent in many different races in numerous countries throughout the World.
